# Seroprevalence of 34 Human Papillomavirus Types in the German General Population

**DOI:** 10.1371/journal.ppat.1000091

**Published:** 2008-06-20

**Authors:** Kristina M. Michael, Tim Waterboer, Peter Sehr, Annette Rother, Ulrich Reidel, Heiner Boeing, Ignacio G. Bravo, Jörg Schlehofer, Barbara C. Gärtner, Michael Pawlita

**Affiliations:** 1 Infection and Cancer Program, German Cancer Research Center (DKFZ), Heidelberg, Germany; 2 Department of Epidemiology, German Institute of Human Nutrition (DIFE) Potsdam-Rehbrücke, Nuthetal, Germany; 3 Institute for Virology, Saarland University Hospital Homburg, Homburg/Saar, Germany; National Cancer Institute, United States of America

## Abstract

The natural history of infections with many human papillomavirus (HPV) types is poorly understood. Here, we describe for the first time the age- and sex-dependent antibody prevalence for 29 cutaneous and five mucosal HPV types from 15 species within five phylogenetic genera (alpha, beta, gamma, mu, nu) in a general population. Sera from 1,797 German adults and children (758 males and 1,039 females) between 1 and 82 years (median 37 years) were analysed for antibodies to the major capsid protein L1 by Luminex-based multiplex serology. The first substantial HPV antibody reactions observed already in children and young adults are those to cutaneous types of the genera nu (HPV 41) and mu (HPV 1, 63). The antibody prevalence to mucosal high-risk types, most prominently HPV 16, was elevated after puberty in women but not in men and peaked between 25 and 34 years. Antibodies to beta and gamma papillomaviruses (PV) were rare in children and increased homogeneously with age, with prevalence peaks at 40 and 60 years in women and 50 and 70 years in men. Antibodies to cutaneous alpha PV showed a heterogeneous age distribution. In summary, these data suggest three major seroprevalence patterns for HPV of phylogenetically distinct genera: antibodies to mu and nu skin PV appear early in life, those to mucosal alpha PV in women after puberty, and antibodies to beta as well as to gamma skin PV accumulate later in life.

## Author Summary

Papillomaviruses (PV) are a large and highly diverse group of DNA viruses that infect cutaneous and mucosal epithelia of warm-blooded vertebrates. Of the more than 100 identified human PV (HPV) types, many cause benign lesions like warts and papillomas, and some also cervical, other anogenital, and oral cancers. For most HPV, transmission routes, pathogenesis, and time and duration of infection are only poorly understood. In the German general population, we investigated the prevalence of antibodies to the capsid proteins of 34 HPV types representative of all five PV genera (alpha, beta, gamma, mu, and nu) that contain HPV. We provide evidence for different age- and sex-dependent seroprevalence patterns of phylogenetically related HPV: antibodies to cutaneous mu and nu PV appear early in life, those to mucosal alpha PV after puberty, and those to beta and gamma skin PV accumulate in adulthood.

## Introduction

Papillomaviruses (PV) are non-enveloped DNA viruses infecting cutaneous or mucosal epithelia of warm-blooded vertebrates. So far at least 118 distinct PV types, more than 100 of them isolated from humans, have been completely described [1]. In addition, about 130 L1 sequence fragments have been isolated by means of a broad spectrum polymerase chain reaction (PCR) representing putatively new cutaneous human papillomavirus (HPV) types [2]. Based on the nucleotide sequence encoding the major capsid protein L1, PV systematics defines 16 genera (sharing less than 60% sequence identity) which encompass 44 species (sharing 60–70% sequence identity).

PV within the same genus may or may not show similar biological and pathological characteristics. Thus, cutaneous HPV are found among the five genera alpha (species 2, 4 and 8), beta (β), gamma (γ), mu (μ), and nu (ν), whereas the 48 HPV types infecting the mucosa belong exclusively to the genus alpha (α). HPV infections are widespread and can cause a variety of mostly benign tumours such as warts and condylomata. However, the infection with certain mucosal HPV types leads to malignant cell proliferation [3]. Fifteen so-called high-risk (HR) and three putative HR mucosal HPV of genus α, most notably the two most prevalent HR types 16 and 18, are found in more than 90% of cervical tumours [4] and with lower frequency in other anogenital and oro-pharyngeal carcinomas [3]. Thirteen of these HPV types have recently been classified as human carcinogens [5]. The mucosal low-risk (LR) HPV types 6 and 11 cause benign genital lesions like condylomata acuminata and low-grade squamous intraepithelial lesions of the cervix. The cutaneous HPV types 1 (genus μ), 2, 3, 10, 57 (genus α) and HPV 4 (genus γ), although belonging to three different genera, are associated with benign plantar, common, and flat skin warts both in the general population and in renal transplant recipients [6],[7]. β PV are found in high copy numbers in benign macular skin lesions of patients with the rare hereditary disease Epidermodysplasia verruciformis (EV) [8]. The same types are also found in non-melanoma skin cancer (NMSC), namely squamous cell carcinoma (SCC) and less frequently basal cell carcinoma (BCC) of the skin, but also in normal skin and plucked hairs of EV patients, immunosuppressed patients, e.g. transplant recipients, and less frequently immunocompetent patients [9],[10]. γ PV and HPV 41 (genus ν) cause benign skin lesions but have been also found in NMSC [1],[3],[11],[12]. Thus, infections with cutaneous HPV are discussed to play a role in the development of NMSC [9],[10].

Besides the extensively studied HPV 16 and some closely related mucosal HR types, only little is known about the natural history of infections by other types. For both HR (e.g. HPV 16, 18, 31, 33, 35, 45, 52, 58) and LR (e.g. HPV 6 and 11) mucosal HPV, transmission occurs mainly via sexual intercourse [13]. However, non-sexual routes of transmission, e.g. oral and perinatal transmission have been reported and cannot be entirely disregarded [14],[15]. The rare disease recurrent respiratory papillomatosis apparently is caused by perinatal transmission of the LR HPV types 6 and 11 [16]. Infections with cutaneous HPV are assumed to occur via skin contact with contaminated material.

For mucosal HPV types, antibodies to the viral major capsid protein L1 have been shown to be markers for present and past infection. Thus serology provides a powerful epidemiological tool to investigate the wide variety of mucosal and cutaneous HPV types and their distribution in the population [17]. DNA detection methods identify only current infections and are restricted to small samples of certain bodily sites. In contrast, for HPV 16 it has been shown that serology may be used as a proxy for lifetime cumulative exposure [17]. However, only 50–60% of HPV 16 DNA positive women develop HPV antibodies and seroconversion can occur even several months after the infection [17],[18]. HPV 16 L1 antibodies have low but stable titres and can be detected decades after the infection [17]. Antibodies to HPV 16 L1 have been shown to be type-specific [17],[18], although some cross-reactivity with closely related types was reported [18],[19]. For the other HPV types, similar characteristics of L1 antibodies are assumed.

Seroepidemiological studies especially on cutaneous HPV infection are scarce and those performed in the past were restricted to a single or few HPV types. To our knowledge, mucosal HPV L1 antibody analyses have been performed so far for HR HPV types 16, 18, 31, 33, 39, 45, 52, 58, 59, 73, for LR HPV types 6 and 11 and for HPV types 13 and 32. The serologically best studied cutaneous types are HPV 1, 5, and 8 [20]–[37], while little or nothing is known about the antibody response to other cutaneous types [38]–[45].

HPV antibody analyses are complex because of the large number of HPV types, and conventional enzyme-linked immunosorbent assay (ELISA) methods allow analysing the reactivity of a serum sample to only one antigen per reaction. We recently developed multiplex serology [46],[47] that allows the simultaneous determination of antibodies to a large number of HPV types and the analysis of more than 1,000 sera per day. Multiplex serology uses viral L1 proteins expressed in bacteria as glutathione S-transferase (GST) fusion proteins as antigens [48],[49]. Rizk *et al.* [50] demonstrated that these recombinant L1 proteins display conformational epitopes. Three first case-control studies on NMSC investigating the antibody response to a total of 16 [39], 31 [45], and 38 [44] different HPV types have been reported. However, our knowledge about the natural history of HPV infections is still limited.

Here we describe for the first time the age- and sex-dependent antibody prevalence for 29 cutaneous and five mucosal HPV types representing 15 species within five phylogenetic genera (α, β, γ, μ, ν) in a general population.

## Results

### HPV antibody reactivity patterns

**Figure 1:**
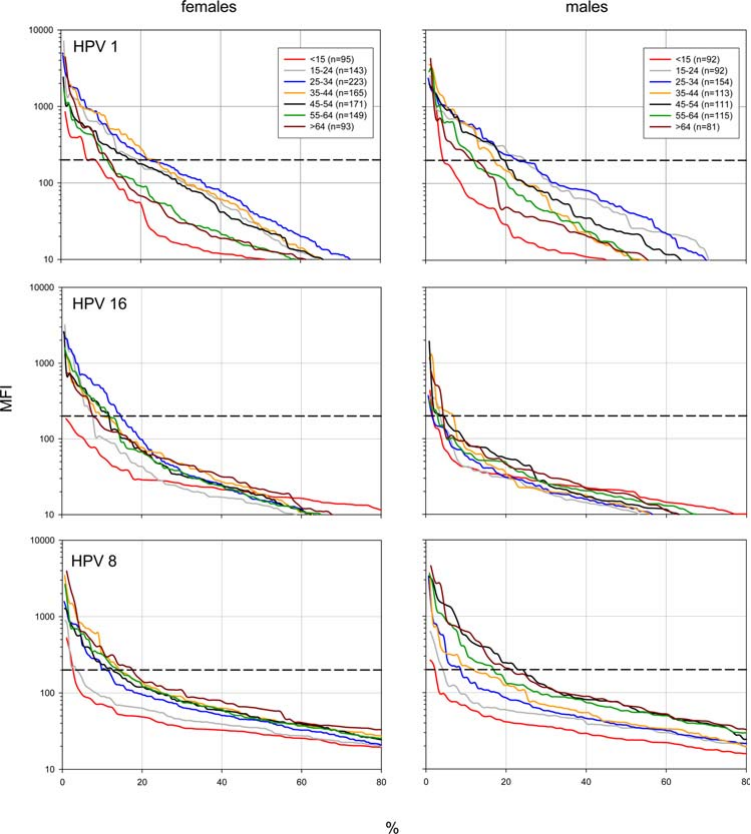
Distribution of antibody reactivity for HPV types 1, 16 and 8 in the German population. Antibodies against HPV L1 proteins were analysed in 1797 sera from the German general population and stratified by sex and age. The strength of the antibody reactions expressed as median fluorescence intensity (MFI) on the y-axis is plotted against the percentile on the x-axis. Data points were connected by spline curves. Colour code, age groups in years and size of the groups are indicated in the inserts. Dashed black lines indicate the cut-off of 200 MFI. The figure reads as follows (upper left panel, HPV 1 antibodies among females): In the 45–54 years age group, approximately 40% showed antibody reactions above 50 MFI, approximately 30% above 100 MFI, and approximately 20% above 200 MFI. For all HPV types and in both genders antibody reactivity was lowest in children. The biggest difference in antibody reactivity was observed between children and young adults (15–24 years) against HPV 1. In comparison to children, antibody levels against HPV 16 were substantially higher in young women but not in men. Antibody reactivity to HPV 8 became stronger with age in both genders and peaked in women at about 40 years and in men at about 50 years. Especially among men, a second peak was found in the oldest age group. These antibody patterns were present also for weak responses well below the chosen cut-off.

To obtain an unbiased (cut-off free) impression of HPV antibodies, the strength of the antibody reactions was plotted against the percentile for each HPV type and age and sex group. Figure 1 shows the plots for HPV types 1, 16 and 8, which are representative for the three major antibody patterns found among the 34 types analysed.

For all HPV types and in both sexes, antibody reactivity was lowest in children. The biggest difference in antibody reactivity was observed between children and young adults (15–24 years) against HPV 1, which is representative for the HPV types within the μ and ν genera. HPV 1 antibody reactivities were similar in adults aged 25–54 years and gradually declined thereafter.

Antibody levels against HPV 16, which is representative for genital/mucosal HPV types within the α PV, changed with age in women but hardly in men. In comparison to children, antibody reactivities were substantially higher in young women and peaked in the 25–34 years age group.

Antibody reactivity to HPV 8, which is representative for HPV types within the β and γ PV, became stronger with age in both genders. A first peak in antibody reactivity was observed in women at about 40 years and in men at about 50 years. Especially among men, a second peak was found in the oldest age group.

These age-dependent antibody patterns were present for both strong and weak responses, down to reactions as low as 50 median fluorescence intensities (MFI).

### HPV antibody prevalence

The analysis of the complex antibody patterns was simplified by the generation of seroprevalence values. HPV seroprevalence is defined here for all HPV types and age and sex strata as percent of sera reacting with a given HPV type above a cut-off value of 200 MFI.

In all 1797 sera, the overall seroprevalence for any of the 34 HPV types analysed was 59.7% (Table 1). However, the overall type-specific seroprevalences strongly depended on age and sex.

**Table 1:** Type-specific age- and sex-stratified HPV antibody prevalence in the German population.

			seropositivity (%)															
								adults										
				children 1–14 years				all adults	15–34 years (young adults)				>34 years (older adults)					
			whole study	children	m	f	f/m		young adults	m	f	f/m	older adults	m	f	f/m	children vs young adults	young adults vs older adults
seropositive for			n = 1797	n = 187	n = 92	n = 95	prev. ratio^a^	n = 1610	n = 612	n = 246	n = 366	prev. ratio	n = 998	n = 420	n = 578	prev. ratio	p^b^	p
**any HPV (n = 34)**			59.7	35.3	34.8	35.8	1.0	62.5	59.8	57.7	62.0	1.1	63.3	63.8	63.8	1.0	****	
**any cut. (n = 29)**			55.5	32.1	29.3	34.7	1.2	58.3	54.7	54.9	54.6	1.0	60.4	62.6	58.8	0.9	****	*
**any muc. (n = 5)**			14.5	4.8	7.6	2.1	0.3	15.6	15.2	11	21	**1.9** **	12.9	12.4	16.4	1.3	****	
**number of types**																		
	**0**		40.3	64.7	65.2	64.2	1.0	37.5	40.2	42.3	38.0	0.9	36.7	36.2	36.2	1.0	****	
	**1**		24.5	24.1	22.8	25.3	1.1	24.5	27.0	27.6	26.0	0.9	23.1	22.1	24.0	1.1		
	**2**		11.5	4.8	6.5	3.2	0.5	12.3	11.6	11.4	12.0	1.1	12.4	12.9	12.5	1.0	**	
	**3**		7.4	4.3	3.3	5.3	1.6	7.8	7.5	5.7	9.3	1.6	7.9	7.4	8.0	1.1		
	**>3**		16.2	2.1	2.2	2.1	1.0	17.9	13.7	13.0	14.8	1.1	19.8	21.4	19.4	0.9	****	**
	**>8**		6.1	0.5	1.1	0.0	0.0	6.7	4.2	3.3	5.2	1.6	7.9	10.0	6.7	0.7	*	**
	**>16**		1.9	0.0	0.0	0.0	n/a	2.2	1.3	0.8	1.6	2.0	2.7	2.9	2.6	0.9		
**type**	**gen.**	**sp.**																
**18**	**α muc.**	7	3.8	1.6	2.2	1.1	0.5	4.0	4.1	2.4	5.2	2.1	4.0	3.8	4.2	1.1		
**16**		9	7.1	0.5	1.1	0.0	0.0	7.8	7.8	2.0	11.7	**5.8** ****	7.8	4.3	10.4	**2.4** ***	****	
**33**		9	4.1	2.1	4.3	0.0	0.0	4.3	5.1	3.7	6.0	1.6	3.9	4.0	3.8	0.9		
**52**		9	1.1	0.0	0.0	0.0	n/a	1.2	0.8	1.2	0.5	0.4	1.4	2.1	0.9	0.4		
**58**		9	4.1	1.1	1.1	1.1	1.0	4.4	6.2	6.5	6.0	0.9	3.3	3.8	2.9	0.8	**	**
**2**	**α cut.**	4	4.1	1.1	1.1	1.1	1.0	4.5	3.1	1.2	4.4	**3.6** *	5.3	6.4	4.5	0.7		*
**57**		4	2.7	1.1	2.2	0.0	0.0	2.9	2.3	0.8	3.3	4.0	3.2	4.3	2.4	0.6		
**3**		2	6.0	8.6	7.6	9.5	1.2	5.7	7.2	6.9	7.4	1.1	4.8	3.8	5.5	1.5		
**10**		2	6.0	3.2	2.2	4.2	1.9	6.3	4.1	3.7	4.4	1.2	7.7	6.0	9.0	1.5		**
**77**		2	6.7	2.1	0.0	4.2	n/a	7.3	6.4	4.1	7.9	1.9	7.8	6.9	8.5	1.2	*	
**any α cut.**		17.1	11.2	9.8	12.6	1.3	17.8	16.0	13.0	18.0	1.4	18.8	18.3	19.2	1.0			
**49**	**β**	3	9.2	1.1	2.2	0.0	0.0	10.1	5.9	6.5	5.5	0.8	12.7	13.3	12.3	0.9	**	****
**76**		3	4.0	1.6	2.2	1.1	0.5	4.3	1.5	1.6	1.4	0.8	6.0	7.1	5.2	0.7		****
**75**		3	5.0	1.1	1.1	1.1	1.0	5.5	2.6	2.4	2.7	1.1	7.2	9.8	5.4	**0.5** **		****
**38**		2	7.6	0.5	1.1	0.0	0.0	8.4	4.6	4.1	4.9	1.2	10.8	12.1	9.9	0.8	**	****
**23**		2	3.5	0.5	1.1	0.0	0.0	3.8	2.0	1.6	2.2	1.3	4.9	6.2	4.0	0.6		**
**9**		2	7.1	2.7	4.3	1.1	0.2	7.6	5.1	4.5	5.5	1.2	9.2	11.4	7.6	**0.7** *		**
**17**		2	7.0	2.7	1.1	4.2	3.9	7.5	3.9	3.7	4.1	1.1	9.7	11.0	8.8	0.8		****
**15**		2	7.1	0.5	0.0	1.1	n/a	7.9	5.1	3.3	6.3	1.9	9.6	11.7	8.1	0.7	**	***
**92**		4	5.2	0.5	0.0	1.1	n/a	5.8	3.4	1.6	4.6	2.9	7.2	8.3	6.4	0.8	*	**
**20**		1	5.5	1.6	2.2	1.1	0.5	5.9	3.9	2.4	4.9	2.0	7.1	8.6	6.1	0.7		**
**93**		1	0.6	0.0	0.0	0.0	n/a	0.7	0.5	0.4	0.5	1.3	0.8	1.2	0.5	0.4		
**24**		1	4.7	0.0	0.0	0.0	n/a	5.2	3.1	1.6	4.1	2.5	6.5	7.4	5.9	0.8	*	**
**5**		1	5.5	1.1	2.2	0.0	0.0	6.0	3.6	2.8	4.1	1.4	7.4	10.5	5.2	**0.5** **		**
**36**		1	3.8	1.1	0.0	2.1	n/a	4.2	3.3	2.4	3.8	1.6	4.7	5.7	4.0	0.7		
**8**		1	11.3	2.1	2.2	2.1	1.0	12.4	7.4	6.5	7.9	1.2	15.4	17.6	13.8	0.8	**	****
**any β**			26.3	11.2	9.8	12.6	1.3	28.0	17.6	16.7	18.3	1.1	34.4	36.9	32.5	0.9	*	****
**95**	**γ**	1	7.7	1.6	2.2	1.1	0.5	8.4	6.4	5.3	7.1	1.3	9.6	10.2	9.2	0.9	**	*
**65**		1	9.9	0.5	1.1	0.0	0.0	11.0	8.3	8.1	8.5	1.0	12.6	14.3	11.4	0.8	****	**
**4**		1	16.9	4.8	6.5	3.2	0.5	18.3	18.3	16.3	19.7	1.2	18.3	20.2	17.0	0.8	****	
**50**		3	4.1	1.1	0.0	2.1	n/a	4.4	2.8	1.2	3.8	3.1	5.4	6.4	4.7	0.7		*
**48**		2	7.4	1.6	1.1	2.1	1.9	8.1	5.7	6.5	5.2	0.8	9.5	10.5	8.8	0.8	*	**
**60**		4	0.7	0.0	0.0	0.0	n/a	0.7	0.3	0.0	0.5	n/a	1.0	1.7	0.5	0.3		
**any γ**			26.8	9.1	9.8	8.4	0.9	28.9	25.2	23.6	26.2	1.1	31.2	34.3	28.9	0.8	****	*
**41**	**ν**		7.4	3.2	2.2	4.2	1.9	7.9	8.8	9.8	8.2	0.8	7.3	10.2	5.2	**0.5** **	*	
**1**	**μ**	1	17.0	6.4	4.3	8.4	1.9	18.2	21.9	23.2	21.0	0.9	15.9	14.8	16.8	1.1	****	**
**63**		2	10.6	2.7	2.2	3.2	1.5	11.5	12.3	13.0	11.7	0.9	11.0	11.4	10.7	0.9	****	
**any μ/ν**		24.7	10.7	8.7	12.6	1.4	26.3	30.2	35.0	27.0	**0.8** *	23.8	24.3	23.5	1.0	****	**	

abbreviations: cut. cutaneous, f females, gen. genus, m males, muc. mucosal, n/a not available, prev. prevalence, sp. species.

a female over male prevalence ratio;

b p, two-sided Fisher's exact test;

*: p<0.05; **p<0.01; ***p<0.001; ****p<0.0001.

HPV types 1 and 4 showed the highest type-specific seroprevalences (17.0 and 16.9%, respectively) (Table 1). Among children, HPV antibody prevalences were mostly low, with the highest seroprevalences for types 3 (8.6%), 1 (6.4%), and 4 (4.8%). Among adults (individuals post-puberty, ≥15 years), seroprevalence was highest again for HPV types 4 (18.3%) and 1 (18.2%), followed by types 8 (12.4%), 63 (11.5%), 65 (11.0%), and 49 (10.1%), and lowest (<1.5%) for types 52, 60 and 93.

**Figure 2:**
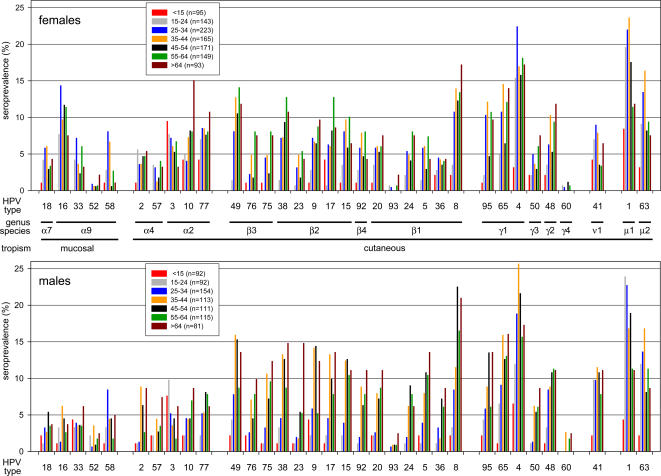
Prevalence of antibodies to 34 HPV types in the German population. Antibodies against HPV L1 proteins of 29 cutaneous and 5 mucosal HPV types were analysed in 1797 sera from the German general population and stratified by sex and age. The type-specific seroprevalences in seven age groups are shown for females and males separately. Colour code, age groups in years and size of the groups are indicated in the inserts. HPV types are specified below each graph, and genus, species, and tropism are shown between the graphs. Seroprevalence patterns were similar for μ and ν PV, for high-risk mucosal α PV in females, and for β and γ PV, respectively. Antibodies to cutaneous μ and ν PV were already present in children with substantial prevalences in young adults (15–24 years). Antibodies to mucosal high-risk HPV appeared after puberty and peaked in the 25–34 years group mainly in women. Seroprevalence for β and γ PV accumulated and peaked late in life in both genders. Most HPV showed two seroprevalence peaks. Age at the first peak varied from about 20 to 50 years for the different genera, but the second prevalence peak rather uniformly occurred at old age, in females at about 60 years and in males at above 64 years.

Seroprevalence patterns were similar for μ and ν PV, for high-risk mucosal α PV in females, and for β and γ PV, respectively (Figure 2). Most HPV showed two seroprevalence peaks. Age at the first peak varied from about 20 to 50 years for the different genera, but the second prevalence peak rather uniformly occurred at old age, in females at about 60 years and in males at above 64 years.

For statistical analysis of the age- and sex-dependent seroprevalence patterns, age groups were combined to increase statistical power (Table 1). Highly significant seroprevalence increases (p<0.0001) from children to younger adults (15–34 years) (but not from younger to older adults >34 years) were seen for mucosal α (HPV 16), γ (HPV 4, 65) and both μ PV (HPV 1, 63). In contrast, none of the 15 β PV types showed highly significant seroprevalence increases from children to younger adults, but 6 types from younger to older adults (HPV 49, 76, 75, 38, 17 and 8). Multiple seropositivity (>3, >8 types) increased significantly (at least p<0.05) from children to younger adults but also from younger to older adults.

The strongest sex difference was observed for the mucosal high-risk HPV type 16. Seroprevalence in younger women was 5.8-fold and in older women still 2.4-fold higher than in men of the same age. For two other mucosal high-risk types, seroprevalence was also slightly elevated in younger women (HPV 18, 2.1-fold and HPV 33, 1.6-fold), however without statistical significance. Seroprevalence for cutaneous α, β and γ PV tended to be higher in younger women than in younger men (median 1.3-fold), but HPV 2 was the only individual type for which this difference was statistically significant. In contrast, women >34 years showed a lower seroprevalence for these genera than men (median 0.8-fold), with significant differences for the HPV types 75, 9, and 5.

### Multiple HPV seropositivity

**Figure 3:**
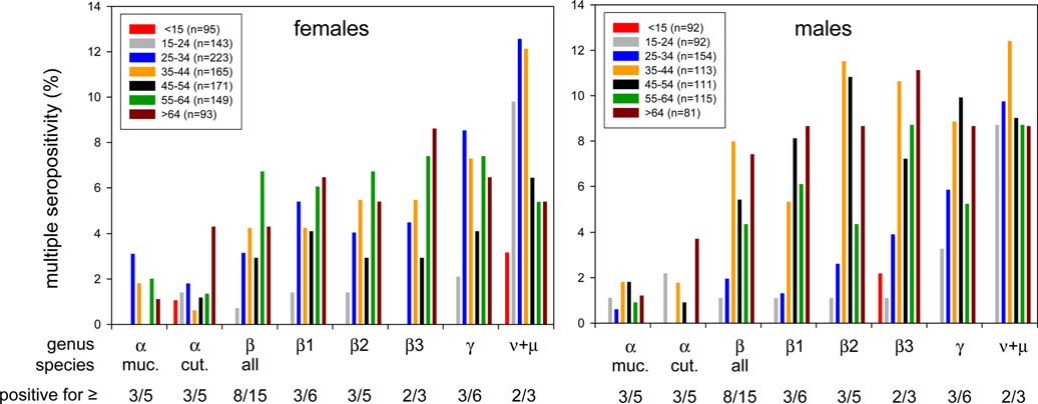
Multiple HPV seropositivity stratified by age and sex. Frequency of multiple seropositivity (seropositivity to at least half of the HPV types analysed within a species or genus) in seven age groups are shown for females and males separately. Colour code, age groups in years and size of the groups are specified in the inserts. Phylogenetic groups (genera and species) and their specific multiple seropositivity definitions (e.g. seropositive for ≥8 out of 15 β PV types) are indicated. muc., mucosal; cut., cutaneous. Multiple seropositivity was lowest for mucosal and cutaneous α PV and in general followed the patterns seen for type-specific seroprevalences (Fig. 2). For μ and ν PV, it was the earliest to occur in young adults of both genders. For mucosal α PV, it was present mainly in women and peaked at 25–34 years. For cutaneous α PV, multiple seropositivity showed an isolated peak in the oldest age group. For β and γ PV, both genders showed two prevalence peaks, in women at around 35 and 60 years and in men some 5 to 10 years later.

Antibody reactivity to more than one HPV type was frequent (Table 1). While 24.5% of the sera (41.0% of the antibody positives) were single positive, 11.5% reacted with two, 7.4% with three and 16.2% with more than three HPV types. Even seropositivities to more than 8 (6.1%) and more than 16 (1.9%) types were observed. Multiple seropositivity (Figure 3), here defined operationally as antibody positivity to at least half of the HPV types analysed within a species or genus, followed in general the patterns seen for type-specific seroprevalences (Figure 2). Multiple seroreactions were rare in children (<15 years) and were most prevalent in the second half of life (>34 years). Mucosal and cutaneous α PV showed substantially less multiple seropositivity than β, γ, μ and ν types.

Multiple seropositvity to μ and ν PV showed a steep increase already in young adults (15–24 years) and thus was the earliest to occur in both genders. Multiple seropositivity to high-risk mucosal α PV in women (but not in men) first occurred and also peaked at 25 to 34 years. For cutaneous α PV, multiple seropositivity showed an isolated peak in the oldest age group in both genders. For β and γ PV, both genders showed two prevalence peaks, in women at around 35 and 60 years and in men some 5 to 10 years later.

## Discussion

Fundamental data on the humoral immune response to HPV infections is scarce. Most serological analyses performed in the past were case-control studies that mainly focussed on mucosal HR HPV types highly prevalent in cervical cancer and mostly on women at productive age. Little is known about the age distribution of HPV infections, especially in children, men and in old-aged individuals, and for many HPV types no serological data is available at all. To understand the natural history of HPV infections, investigations of a broad range of HPV types belonging to different species and genera are needed. This is the first large study to analyse simultaneously antibody reactions to 34 HPV types in a general population. The cross-sectional data presented here allow a detailed and comprehensive assessment of the seroprevalence in the adult German general population by HPV type (and higher taxonomic order), sex and age. The data for the children originating from two hospitalized groups may be less representative for the German population. The conclusions of our study results might also be limited due to the use of unadjusted seroprevalence values. However, when age standardization was applied seroprevalence estimates changed only marginally.

HPV of the same genus, with the exception of cutaneous α PV, showed similar antibody patterns and are therefore discussed as group. For comparisons with previously published prevalence data across different laboratories it is important to keep in mind that different assay formats and, probably even more important, different cut-off definitions may greatly influence absolute prevalence figures.

Genus μ PV are associated with distinct common warts, HPV 1 with deep palmoplantar warts (myrmecia) and HPV 63 with cystic or punctate, mainly plantar warts [6]. Warts [51],[52] and especially HPV 1 positive warts mostly occur at the age of 5 to 20 years [6], [28], [29], [53]–[55] while patients with HPV 63-induced warts were 10 to 39 years old [54],[56]. HPV 41 of genus ν originally isolated from a facial wart of a 15 years old girl was found mainly in plane hand warts but also in some skin carcinomas and actinic keratoses [1],[3],[11],[12].

In our study, seroprevalence for HPV 1 (17.0%) was the highest among all types analysed, antibodies to HPV 63 (10.6%) were less prevalent. These findings are in agreement with a study reporting HPV 1 as the most prevalent type in warts from Germany (27.3%) [57] and with other non-German studies reporting higher prevalences in warts for HPV 1 (44.1% and 31.5%) than for HPV 63 (0.9% and 16.4%), respectively [54],[58]. Serological studies using virus particles purified from warts as antigens also frequently found antibodies to HPV 1 among mostly adults in 10% [30] and 19.3% [20] of the control sera. However, HPV 1 antibody prevalences of 32% and higher in both patients with and without a history of foot warts have been reported [28]–[34],[36]. The substantial seroprevalence found in all studies indicates a high frequency of infections with HPV 1 in the normal population. In addition, HPV 1-induced warts contain high viral loads [53],[59] likely to result in more antigen presentation and thus in increased antibody production.

In both sexes, antibodies against μ and ν PV were the first to be seen. Seroprevalence peaked after the age of 14 and declined only slightly thereafter. In line with this observation, Hamsikova *et al.* [37] and Pfister *et al.* [28] reported a seroprevalence peak for HPV 1 at 13–30 years and 11–20 years, respectively. Two other studies reported seroprevalence to peak in children younger than 15 years [29],[35]. Assuming no newly acquired foot wart in adults and thus no new seroconversion, the data suggest a delayed but long lasting antibody induction detectable even decades after the initial infection.

In our study, type-specific seroprevalences for HR mucosal HPV in adult women (>14 years) were highest for HPV 16 (10.9%) followed by HPV 33 (4.7%), 18 (4.6%), 58 (4.1%), and 52 (0.7%). DNA prevalences for HR types in German women without cervical abnormalities were concordantly also highest for HPV 16 (14.6%) followed by HPV 58 (2.7%), 52 (2.5%), 33 (2.2%), and 18 (1.7%) [60]. Another study on type-specific HPV DNA prevalence in West German women also found HPV 16 (26.2%) as the most frequent type, followed by HPV 31 (10.1%), 18 (5.3%), 58 (4.5%), 33 and 52 (both <4%) [61]. In adult men, we found the highest seroprevalence for HPV 58 (4.8%), inconsistent with DNA data identifying HPV 16 as the most prevalent HR type in men [62].

Antibodies to HR mucosal α PV were rare in children (HPV 16 0.5%, other HR types 0.0–2.1%), which is consistent with seroprevalence rates for HPV 16 in children ranging from 1.5–7.6% reported by other studies [63]. In women, these antibodies increased after puberty (reflecting the start of sexual activity) and peaked at 25–34 years, whereas in men seroprevalence did not increase strongly until the second half of life. This antibody increase and peak in young women was reported by several other studies [64]–[71] and is in agreement with a DNA prevalence peak for mucosal (predominantly HR) HPV frequently found in women younger than 30 years. With increasing age, HPV DNA prevalence decreases [72]–[76], however a second peak in women around 50 years [77] or 60 years and older [78]–[81] has been reported. Consistently with the latter studies, we found a second seroprevalence peak for HR HPV in women older than 45 years.

HPV 16 serology in comparison to the other HR HPV was unique. Only for HPV 16, seroprevalence was significantly higher in women than in men both among younger and older adults, while seroprevalence for the other mucosal HR types showed only a non-significant increase in women. Although studies on HPV seroprevalence in men are rare, the lower seroprevalence for mucosal HPV types 6, 11, and 16 assessed by virus-like particle (VLP) serology is well known [66], [71], [82]–[89]. Several biological and anatomical sex-dependent differences were discussed as possible explanations: HPV infections of the penis involve rather keratinized than mucosal epithelium which might be less susceptible for the virus, less productive, and less accessible for the immune system. Men might have rather transient infections with lower viral loads that induce a weaker antibody response than in women. Overall DNA prevalence of mucosal HPV types in cytologically normal cervical smears ranges from 1.4% to 44% [90],[91]. In healthy men, overall HPV DNA prevalence values of 3.5% to 45% for mucosal types in exfoliated cells of the penis are in the same range [62]. In view of this similar prevalence in penile versus cervical samples, the assumption of lower susceptibility of men cannot be held up.

It has been shown that persistent infection is associated with higher seroprevalence [92],[93]. Thus, HPV 16 might not only be more prevalent in women compared with men but also more persistent, with higher viral DNA loads, and with a greater amount of intact virions. HPV 16 is the most prevalent HPV type in low-grade squamous intraepithelial lesions [94], and these lesions are known to express L1 [95]. In addition, HPV 16 is possibly more immunogenic than other HR types [96],[97].

The most heterogeneous antibody distribution with regard to both age and type was seen for cutaneous α PV, although they are closely sequence-related. However, due to the overall low seroprevalence, the power of this pattern analysis is low. While antibodies to HPV 3 were frequent in children and decreased with age, seroprevalences for HPV types 2, 57, 10, and 77 were low in children and increased with age. These findings may suggest different natural histories of the individual wart-associated α PV types. HPV 3 and 10 are associated with plane, HPV 2 and 57 with common, and HPV 27 with intermediate warts [6]. HPV 77 has been found in tumours and warts of immunosuppressed but not in lesions of immunocompetent individuals [98]. HPV 2-associated warts have been most frequently found in the 20–40 years age group [53],[54],[99], and in another study the prevalence of HPV 2/27/57-induced warts peaked at the age of 21–25 years [55].

For β and γ PV, age distribution patterns were very homogeneous. Seroprevalence for these types was low in children and increased with age. In middle-aged women, seroprevalence tended to be higher than in men of the same age. In older adults, this sex ratio shifted to a higher seroprevalence for men. We observed two seroprevalence peaks, both occurring slightly earlier in women than in men. At present, we can only speculate about potential reasons for the sex-associated serological differences for β and γ PV. In men, body hair is more abundant, the use of chemicals on the skin (cosmetics) is probably reduced, and for the population studied here sun exposure for large parts of the body during outdoor work might have been more frequent.

Serological studies showed elevated prevalences of antibodies to β PV, mainly HPV 5 and 8, in EV and in immunosuppressed patients, in patients with dermatological diseases like squamous cell carcinoma of the skin [20], [23]–[25], [38]–[41] or psoriasis [21],[24] and in patients with second degree burns [20]. Feltkamp *et al.* found a statistically significant association of seropositivity with age and male sex for HPV 24 but not for HPV 5, 8, 15, 20 and 38 [38] while in two other studies HPV 8 seropositivity was not correlated with age and/or sex [23],[24].

DNA prevalence data indicate an ubiquitous distribution of these types in the population. Cutaneous HPV mainly of genus β are found in normal skin and plucked hairs of different body sites from healthy individuals, with up to 96% overall DNA prevalence in adults [10],[100],[101]. In samples from different skin regions of an individual, the same types are frequently found [100],[102]. Type patterns in plucked eye brow hairs and on healthy skin of an individual frequently persist [101],[103]. DNA from β and γ PV has been found in specimens taken with wet cotton-tipped swabs from the foreskin of infants within days after birth [104] thus demonstrating exposure. However, in the absence of additional data showing active virus infection in the infant's cells, it remains unclear whether these findings show infection to occur early in life. In adults, HPV DNA prevalence increases with age in both genders [100],[105]. We observed increasing seroprevalence in females until around 40 and in males until around 50 years. This suggests that seroconversion for HPV of these two genera in contrast to μ and ν PV is extremely slow and occurs only years or even decades after the initial infection. Alternatively, this might indicate that despite early exposure active infection occurs much later. DNA of these types in immunocompetent individuals is present only in very low copy numbers [106]. A single contact with these types might therefore not result in antibody induction. However, accumulation of cutaneous HPV infections over lifetime due to an increased exposure via intense skin contacts could lead to higher overall viral loads and thus to an increasing seroprevalence with age. In addition, the weakening of the immune system in older age and/or perhaps increased sun-exposure might be responsible for the inability to control viral replication which consequently leads to higher viral loads and higher seroprevalences.

For most of the analysed HPV types, seroprevalence peaked twice. A possible explanation for the second peak at ages beyond 55 years is a reactivation of latent infections perhaps by reduction of immune surveillance with increasing age followed by increasing viral load and antibody induction. For mucosal α PV in women, it might in addition be caused by changes in the genital epithelium associated with menopause. Alternatively, the seroprevalence in older age groups of this serum collection might be due to a cohort effect. Possibly varying behaviour in different birth cohorts might have influenced exposure, such as sexual behaviour for genital HPV. We speculate that also environmental factors, such as sun exposure or nutrition, may have influenced the extent of HPV replication and/or the functionality of the immune system. The sera of this study were collected in 1987 and 1988. It remains to be examined whether this age-dependent distribution will also be present in sera collected 20 years later.

We observed a substantial frequency of multiple seropositive reactions, e.g. 48% of HPV 5 positive sera reacted also with HPV 8. Multiple infections with mucosal HPV [62],[91] and even more often with cutaneous HPV both in immunosuppressed as well as immunocompetent individuals are common [100],[107]. Multiple seropositivity may result from type-specific reactions to multiple infections and/or could be due to cross-reactive antibodies induced by an infection with only one or few HPV types. The antibody detection assay used here is not able to distinguish these two possibilities. To address this issue, we investigated i) whether double seropositivity for a given pair of HPV types was observed more often than expected by chance and ii) whether double seropositivity was correlated with sequence identity of the respective L1 proteins (shown in supplementary material Figure S1 and Text S1). If multiple seropositivity was due to cross-reactivity, it should be explainable by the degree of relatedness of HPV types. Our results showed that multiple seropositivity was not or only weakly correlated with L1 amino acid sequence relatedness, and thus we consider the measured antibodies to be mainly type-specific. However, cross-reactivity can only be appropriately investigated by means of absorption experiments or monospecific antisera which are currently not available for most HPV types.

One limitation of this study is the use of a uniform, arbitrarily defined cut-off for seroprevalence calculations. For assays of antibodies to sexually transmitted genital HPV, cut-off definitions can be based on seroreactivities in groups of virgins [108] while for the cutaneous types representing the vast majority of HPV types analysed here the definition of a mostly uninfected and thus mostly seronegative group is not possible. In the absence of defined reference sera that could be used as international standards, any cut-off definition has an arbitrary component and seroprevalence values obtained by different laboratories in general should only be compared with caution.

In our study all antigens were identically constructed L1 fusion proteins expressed in the same bacterial expression system. The full-length L1 fusion protein density on the beads for different HPV types was very similar, since MFI values obtained after staining of the carboxy (C)-terminal tag epitope with a monoclonal antibody varied less than two-fold. Thus, given the similar properties of the antigens the use of an uniform cut-off appears justified. To avoid false-positivity by low-level cross-reactivity and to increase type specificity of the seroprevalence values, a cut-off well above background levels was chosen. Thus, the cut-off is rather stringent and probably underestimates the true seroprevalence of cutaneous HPV infections.

However, as shown in Figure 1, the major conclusions are independent of the chosen cut-off. The age-dependent distribution of skin HPV antibodies as well as the unique pattern of HPV 16 antibodies in women are also present at very low seroreactivity levels (MFI values well below the chosen cut-off).

Direct comparison of GST-L1 fusion protein- and VLP-based ELISA with human sera has been performed for HPV 16 and 18, and showed good correlation [48],[50]. Of 46 monospecific monoclonal antibodies detecting conformational epitopes on VLP of 9 mucosal types of which 4 (HPV 16, 18, 33, and 52) are also used here all reacted with the GST-L1 fusion protein of the same type [50] demonstrating the ability of GST-L1 to bind type-specific antibodies. Based on these observations, it is reasonable to assume similar properties for the other 30 HPV types investigated here. We cannot entirely exclude cross-reactivity and thus unspecific antibody reactions. Like VLP, GST fusion proteins present conformational and neutralising epitopes but display a higher amount of linear/cross-reacting epitopes than VLP [50]. Thus, under the condition that only highly purified, intact VLP preparations are used as antigens, VLP-based assays may yield a higher ratio of specific to unspecific reactions than with GST-L1-based serology.

In conclusion, the serological results presented here suggest different seroprevalence patterns of phylogenetically related HPV: Antibodies to cutaneous μ and ν PV appear late in childhood, those to mucosal high-risk HPV after puberty mainly in women, and seroprevalence for β and γ PV peaked late in life in both genders.

The assays developed here allow further serological investigations to enlarge our still scarce knowledge on the natural history of especially cutaneous HPV infections. Interesting issues are e.g. effect of sun exposure on HPV prevalence, and the immune response in EV patients and patients with cutaneous warts in comparison to healthy people. In addition, seroepidemiological case-control studies might help to understand the potential role of cutaneous HPV in the development of non-melanoma skin cancer.

## Materials and Methods

### Recombinant HPV L1 proteins

HPV L1 open reading frames were expressed via the pGEX4T3 vector (GE Healthcare, München, Germany) in *E.coli* Rosetta cells (Merck, Darmstadt, Germany) as double fusion proteins with amino (N)-terminal GST and a C-terminal peptide (tag) consisting of the last 11 amino acids from the large T antigen of simian virus 40. The expression constructs for the L1 proteins of HPV types 16 and 18 have been described [48]. Constructs for full-length L1 of HPV types 1a, 2a, 3, 4, 5, 8, 9, 10, 15, 17, 20, 23, 24, 33, 36, 38, 41, 48, 49, 50, 52, 57, 58, 60, 63, 65, 75, 76, 77, 92, 93, and 95 were generated in the same fashion. HPV genomes used as PCR templates, amplified regions, and cloning enzymes are listed in Table 2. HPV sequences were verified by commercial sequence analyses and aligned by HUSAR (Heidelberg Unix Sequence Analysis Resources, http://genome.dkfz-heidelberg.de/). Sequences of 27 of the 34 L1 constructs were identical with the published HPV nucleotide sequence. Seven of the 11 sequence variations in L1 of HPV 5, 8, 10, 16, 18, 41, and 57 resulted in amino acid changes (Table 2). Eight of the mismatches were present already in the parental plasmids, indicating possibly mistakes in the published sequences. Of the three PCR-generated mismatches, only one lead to a threonine (T) to serine (S) amino acid change in HPV 10 L1. This error was not corrected since T and S are functionally very similar amino acids. The sequence of the parental HPV 5 L1 ORF used here [109],[110] was newly determined and submitted to GenBank (AM922325). In comparison to the most closely related published HPV 5 sequence (HPV 5b [111], D90252), it showed 17 mismatches and a 27 nucleotide deletion (2.9% sequence variation) and therefore probably represents a new subtype.

**Table 2:** Characteristics of expressed HPV L1 sequences.

HPV type	Accession number^a^	Amplified region/cloning enzymes^b^	Mismatches^c^
1a	V01116	B-5431-6936-S	
2a	X55964	Sm-5742-7271-S	
3	X74462	B-5751-7262-S	
4	X70827	B-5345-6892-S	
5	AM922325	B-1-1551-S	G1533A^d^,^e^
8	M12737	B-5851-7392-S	A7383G^d^,^e^
9	X74464	B-5745-7265-S	
10	X74465	E-5823-7331-S	C7324G (T to S)^d^
15	X74468	B-5703-7223-S	
16R^f^,^g^	not yet entered^h^	Sm-5669-7153-S	A6434G (T to A)^i^
17	X74469	B-5724-7244-S	
18^f^,^g^	X05015	Sm-5643-7133-S	C5701G (P to R)^i^, C6460G (P to R)^i^, C6625G (P to R)^i^, C6842G^e^,^i^
20	U31778	B-5893-7440-S	
23	U31781	Sm-5660-7177-S	
24	U31782	B-5713-7248-S	
33	M12732	B-5594-7090-S	
36	U31785	Sm-5891-7438-S	
38	U31787	Sm-5661-7190-S	
41	X56147	E-5546-7084-S	T5905C^e^,^i^, G6165C (S to T)^i^
48	U31789	E5202-6740-S	
49	X74480	B-5811-7337-S	
50	U31790	E-5231-6775-S	
52	X74481	B-5643-7151-S	
57	X55965	B-5751-7232-S	T6180A (Y to N)^i^
58	D90400	B-5643-7136-S	
60	U31792	E-5395-6918-S	
63	X70828	E-5371-6891-S	
65	X70829	B-5326-6873-S	
75	Y15173	B-5771-7297-S	
76	Y15174	Sm-5799-7325-S	
77	Y15175	B-5789-7297-S	
92	AF531420	B-5638-7173-S	
93	AY382778	B-5640-7178-S	
95	AJ620210	B-5368-6912-S	

a source of genome sequences: GenBank.

b B = BamHI, E = EcoRI, S = SalI, Sm = SmaI; first and last HPV nucleotide.

c nucleotide changes and positions, amino acid changes are indicated in brackets.

d PCR-generated mutation.

e silent mutation.

f described in Sehr *et al.* 2002 [48].

g L1 lacks 10 N-terminal amino acids.

h ftp://ftp-t10.lanl.gov/pub/papilloma/GenBank-files/Human-papilloma/HPV16R.gb.

i mutations present in parental HPV plasmid.

Fusion protein expression was induced at room temperature by 0.25 mM isopropyl-β-D-thiogalactoside (IPTG) and six h after induction bacteria were harvested. The pellet from a 1 liter culture was resuspended in 10 mL of 40 mM Tris, 200 mM NaCl, 1 mM EDTA, pH 8.0, 2 mM DTT containing complete protease inhibitor cocktail (Roche, Mannheim, Germany), and lysed with a high pressure homogenizer (Avestin, Ottawa, Canada). After incubation with 2 mM ATP and 5 mM MgCl_2_ for 1 h at room temperature, lysates were cleared from insoluble components (e.g. cell membranes) by centrifugation (14,000 rpm, 4°C, 30 min). For all HPV types, the proportion of insoluble fusion protein was low. Supernatants were stored with 50% glycerol at −20°C.

Fusion protein expression was characterised by Coomassie-stained sodium dodecyl sulfate polyacrylamide gel electrophoresis (SDS-PAGE) as described [49]. Western blot analyses using GST and tag specific antibodies showed full-length fusion proteins of approximately 84 kDa and some additional minor bands of shorter fragments. N-terminal (GST-reactive) fragments were more prevalent than C-terminal (tag-reactive) fragments indicating incomplete translation rather than proteolysis as cause for the smaller fragments (data not shown).

Verification and concentration of full-length GST-L1-tag proteins in the lysates was determined by GST-capture ELISA [49] using a tag-specific antibody for fusion protein detection. For all constructs, glutathione-casein saturation with fusion protein was reached with 200 µg total lysate protein/mL or less. Half maximal binding ranged from 16.5 µg/mL (HPV 3 L1) to 0.3 µg/mL (HPV 48 L1) (median 2.6 µg/mL) indicating about 55-fold variation in GST-L1-tag concentrations in the lysates.

### Human sera

Anonymous sera from 1797 individuals (age 1–82 years, median 37 years; 758 males and 1039 females) were analysed. These sera originated from three serum collections. The largest contribution is from the population-based German Nutrition Survey (Nationale Verzehrstudie NVS, [112],[113]) collected in the German population aged 4–94 years between October 1985 and January 1989 (n = 23,209). The VERA study (Verbundstudie Ernährungserhebung und Risikofaktorenanalytik) is a random subsample of the NVS population aged 18 years and older collected in 1987 and 1988 (n = 1988) [112],[113]. We tested the available 1573 (79.1%) VERA serum samples (age 18–82 years, median 41 years; 651 males and 922 females). Sera from children (below 19 years) were obtained from two other collections: (i) 175 sera from children (age 2–18 years, median 9 years; 71 males and 104 females) treated in 2002 at the university hospital in Homburg (Germany) were randomly selected from a collection of stored diagnostic sera and (ii) 49 sera (age 1–10 years, median 5 years; 36 boys and 13 girls) collected for a previous HPV serology study [114] in 1991 and 1992 from children treated at the university hospital in Heidelberg (Germany).

The age structure of the tested VERA samples is indifferent from the age structure of the NVS population aged 18 years and older (p = 0.9808), and the age structure of our total study (1573 sera from VERA, 175 children sera from Homburg, and 49 children sera from Heidelberg, total n = 1797) is characteristic for the age structure of the total NVS population (p = 0.9629). This allows the conclusion that our study is likely to be representative of the German population.

### Multiplex serology

Sera were analysed simultaneously for antibodies to 34 HPV types by multiplex serology as described [46],[47]. Briefly, GST-L1-tag fusion proteins from cleared lysates were affinity-purified *in situ* through binding to the glutathione casein-coated fluorescence-labelled polystyrene beads. Each fusion protein was bound to a spectrally distinct bead set. A total lysate protein concentration of 1 mg/mL was used for all lysates, an at least five-fold excess to saturate GST fusion protein binding on the beads. Monoclonal anti-tag antibody binding to the different antigen-loaded bead sets varied less than two-fold, indicating similar full-length L1 fusion protein density for the different HPV types. Fusion protein-loaded bead sets were mixed. Sera were pre-incubated at 1∶50 dilution in PBS containing 1 mg/mL casein, 2 mg/mL lysate from bacteria expressing GST-tag alone to block antibodies directed against residual bacterial proteins and GST-tag, 0.5% polyvinylalcohol (PVA, Sigma-Aldrich), 0.8% polyvinylpyrrolidone (PVP, Sigma-Aldrich) and 2.5% Superchemiblock (Millipore, Billerica, MA, USA) to suppress unspecific binding of antibodies to the beads themselves [47]. Serum dilutions were incubated with the same volume of mixed bead sets, resulting in a final serum dilution of 1∶100. Bound antibodies were detected with biotinylated goat-anti-human IgG (H+L) secondary antibody and streptavidin-R-phycoerythrin. A Luminex analyser (xMAP, Luminex Corp., Austin, Texas, USA) was used to identify the internal colour of the individual beads and to quantify their reporter fluorescence (expressed as median fluorescence intensity (MFI) of at least 100 beads per set per serum). A fusion protein consisting of GST and tag without intervening viral antigen served for background determination.

### Assay design and data processing

The glutathione-casein coupled bead sets were loaded with their respective antigen in one batch. The efficiency of antigen loading on beads was quantified via the C-terminal tag as described [46]. Values (mean MFI of 3 wells) for the different antigens ranged from 4604 (HPV 58 L1) to 9065 (HPV 95 L1) with a median of 6180 MFI. Correct antigen loading was verified with 25 reference sera with known HPV antibody pattern. These sera originated from two earlier studies analysing the antibody reactivity to 16 [39] and 31 HPV types [45] by multiplex serology. All types except HPV 33, 52, 58, and 60 were represented in the reference sera.

Study sera were analysed once on three consecutive days. A quality control panel (QC) of 46 sera was included each day resulting in three QC data sets to determine inter-day variation. For inter-day variations of raw MFI values, Pearson correlation coefficients (R^2^) for the individual antigens ranged from 0.761 to 0.986 (median 0.963) for day 2 versus 1 and from 0.691 to 0.989 (median 0.947) for day 3 versus 1. The raw data of days 2 and 3 for each antigen were divided by the slopes of the regression lines of the QC data pairs of days 1 and 2 or days 1 and 3, respectively, to correct for inter-day variation.

The QC sera were also pooled and included as positive standard on each plate. Inter-plate variation coefficients (CV) calculated from these data for the various antigens ranged from 13.0% to 23.4% with a median of 16.6%.

Auto-fluorescence of each bead set and background reactions resulting from binding of secondary reagents to the antigen-loaded beads were determined in one well per plate without human serum. After correction for inter-day variation, mean background values (range 4 to 27 MFI, except for unusually high bead backgrounds of 150 and 350 MFI for HPV 4 L1 and HPV 33 L1, respectively) were subtracted from the raw MFI values and then antigen-specific reactivity was determined by subtraction of the MFI of GST-tag from the MFI of the specific antigen. Cut-off values to define seropositivity for all antigens were arbitrarily set to 200 MFI.

### Statistical analysis

Serological data were stratified by sex and age. Since sexual transmission is known to play a major role in mucosal HPV infection, the first age group encompassed children (14 years and younger), followed by age groups in 10 year intervals. The age distribution of our study and the German standard population as reported by [115] is similar (p = 0.695), and we present data stratified by age. Age standardization was applied but changed seroprevalence estimates only marginally. Therefore, unadjusted seroprevalences are reported throughout this manuscript. Statistical significance of differences in seroprevalence was assessed by two-sided Fisher's exact test. P-values below 0.05 were considered statistically significant.

## Supporting Information

Figure S1 Double seropositivity in relation to amino acid sequence identity of the paired HPV L1 proteins
(0.57 MB TIF)

Text S1 Double seropositivity in relation to amino acid sequence identity of the paired HPV L1 proteins
(0.03 MB DOC)
