# Supplementary figures and images for: Seroprevalence of 34 Human Papillomavirus Types in the German General Population
Source: PLoS Pathog. 2008 Jun 20;4(6):e1000091. doi: 10.1371/journal.ppat.1000091 (PMC2408730; doi:10.1371/journal.ppat.1000091)

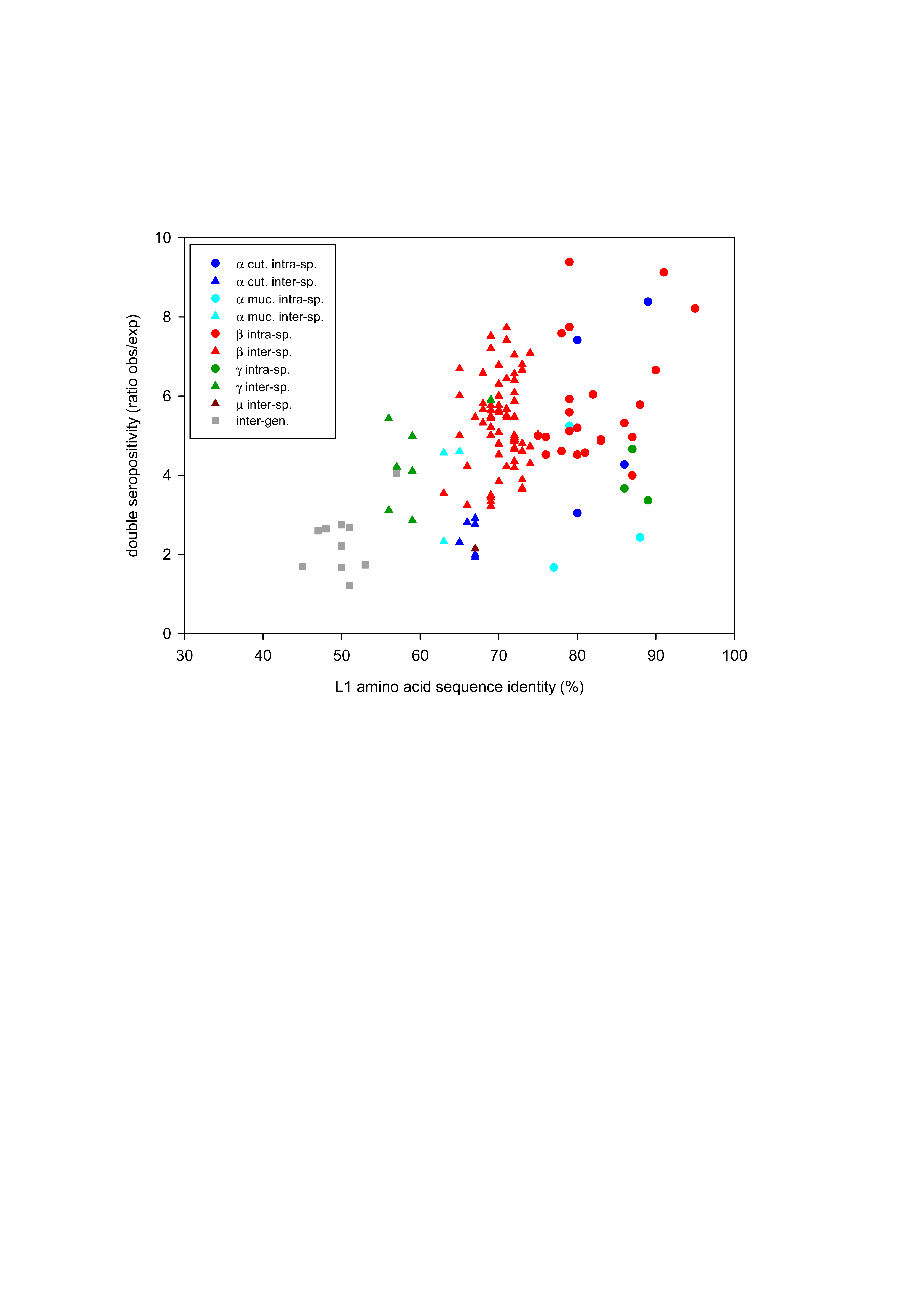

Supplement: Figure S1 — Double seropositivity in relation to amino acid sequence identity of the paired HPV L1 proteins (0.57 MB TIF) [file ppat.1000091.s001.tif]
