# Supplementary material for: Seroprevalence of 34 Human Papillomavirus Types in the German General Population
Source: PLoS Pathog. 2008 Jun 20;4(6):e1000091. doi: 10.1371/journal.ppat.1000091 (PMC2408730; doi:10.1371/journal.ppat.1000091)
Supplement: Text S1 — Double seropositivity in relation to amino acid sequence identity of the paired HPV L1 proteins (0.03 MB DOC) [file ppat.1000091.s002.doc]

**Double seropositivity in relation to amino acid sequence identity of the paired HPV L1 proteins**

We investigated i) whether double seropositivity for a given pair of HPV types was observed more often than expected by chance and ii) whether double seropositivity was correlated with sequence identity of the respective L1 proteins. The amino acid (aa) sequences of the expressed HPV L1 proteins described in Table 2 were aligned with CLUSTALW (http://www.ebi.ac.uk/Tools/clustalw/), and pairwise sequence identities were calculated. For pairs of HPV types, the expected double seropositivity was calculated as the product of the observed individual seroprevalence values and the ratio of observed over expected double seropositivity was plotted against the amino acid sequence identity of the corresponding pair of HPV L1 proteins (Figure S1). The analysis was restricted to high prevalence age groups, encompassing individuals with 25-44 years for μ types (both sexes), 25-44 years for α mucosal types (females only) and >54 years for α cutaneous, β and γ types (both sexes). HPV 52, 60, and 93 were excluded due to low overall seroprevalence. Inter-genus analysis was restricted to individuals with 25-44 years and to the prevalent HPV types 16, 8, 95, 41 and 1 as representatives of each genus. Colour and symbol code is indicated in the insert. gen., genus; sp., species; cut., cutaneous; muc., mucosal; obs, observed; exp, expected.

The median sequence identity among inter-genus pairs was 50%. Among inter-species pairs, it was lowest for γ PV (59%), followed by mucosal α (64%), cutaneous α (67%), μ (67%), and β PV (70%). Intra-species pair identity ranged from 75% to 95% (median 81%). Pairs within α PV species 4, β PV species 3, and γ PV species 1 showed identities of at least 86%, with median values of 89%, 90% and 87%, respectively, in contrast to median values of 79% to 81% for the other α and β PV species.

For all pairs, observed double seropositivity was more frequent than expected (median ratio observed over expected 5.0, range 1.2 to 9.4). The ratio was lowest for inter-genus pairs (median 2.4, range 1.2 to 4.1). Ratios for inter-species pairs were higher among mucosal α (median 4.6, range 2.3 to 4.6) and γ PV (median 4.2, range 2.9 to 5.9) than among cutaneous α PV (median 2.5, range 1.9 to 2.9), although the median sequence identity among γ (59%) and mucosal α PV (63%) was lower than among cutaneous α PV (67%). β PV (median sequence identity 70%) showed the highest observed over expected ratios (median 5.5, range 3.2 to 7.7).

When plotting ratios versus sequence identity, the initial overall impression was that higher degrees of sequence identity indeed resulted in increased observed over expected ratios (Figure S1). However, within a given genus, double seropositivity appeared not to be correlated with sequence identity. Inter-genus pairs showed a correlation (R² for linear regression) of 0.22, i.e. only 22% of the variation in the ratio of the inter-genus pairs could be explained by variation in sequence identity. This correlation was mainly driven by the HPV 8/95 pair that showed the highest sequence identity within this group (57% versus 45-53% for the other pairs) and a ratio of 4.1. Without this pair, the correlation for the inter-genus pairs was only 0.03. For β PV intra- and inter-species pairs, there was very little or no correlation (R² 0.10 and 0.003, respectively). For γ PV intra- and inter-species pairs, the correlation was somewhat higher (R² 0.17 and 0.28, respectively), but for the inter-species pairs it was driven by the HPV 48/50 pair, which exhibits a much higher amino acid sequence identity (69%) than the other pairs (56-59%). Without this pair, the correlation was 0.02. The highest degree of correlation was observed for inter-species pairs of mucosal α PV and for intra-species pairs of cutaneous α PV (R² 0.26 and 0.18, respectively). However, for intra-species pairs of mucosal α PV (R² 0.02) and inter-species pairs of cutaneous α PV (R² 0.01), the correlation was very weak and slightly inversed, i.e. we observed lower ratios with increasing amino acid identity.

Double seropositivity ratios for intra-species pairs were not substantially higher than for inter-species pairs, although the latter are much less closely related to each other. While for the two cutaneous α PV species the intra-species double seropositivity ratio was slightly higher (median 5.8) than the inter-species ratio (median 2.5), intra-species ratios remained at the same level of the inter-species ratios for β PV (5.2 versus 5.5), and were even lower for γ (3.7 versus 4.2) and mucosal α PV (2.4 versus 4.6). The intra-species ratios varied strongly (range 1.7 to 9.4, median 5.0) and were mostly independent of sequence identity. For the α PV species 2 types 10 and 77 (80% identity), the double seropositivity ratio was 7.4, but for the pairs 3 and 10 (86% identity) as well as 3 and 77 (80% identity), the ratios were 4.3 and 3.0, respectively. For β PV species 1, the HPV pair 5 and 8 (87% identity) had a ratio of 5.0, while the less closely related pairs 20 and 24 as well as 20 and 36 (both <80% identity) showed ratios of 7.6 and 9.4, respectively. For these pairs, sequence identities were calculated also only for the combined surface-exposed L1 loop regions BC (aa 50-61, numbering according to the HPV 16 L1-structure [1]), DE (aa 132-150), FG (aa 260-294), and HI (aa 346-359). The observation that intra-species double seropositivity ratios are not correlated with sequence identity held also true when instead of the whole L1 sequences only the surface-exposed loop regions were used to calculate sequence identity.

Taken together, these results suggest that the observed double and multiple positive reactions are unlikely to be mainly due to cross-reactivity and rather reflect multiple infections of related HPV types.

**Reference**

1. Chen XS, Garcea RL, Goldberg I, Casini G, Harrison SC (2000) Structure of small virus-like particles assembled from the L1 protein of human papillomavirus 16. Mol Cell 5: 557-567.
